# Starch Formates: Synthesis and Modification

**DOI:** 10.3390/molecules26164882

**Published:** 2021-08-12

**Authors:** Sascha Blohm, Thomas Heinze, Haisong Qi

**Affiliations:** 1 Centre of Excellence for Polysaccharide Research, Institute for Organic Chemistry and Macromolecular Chemistry, Friedrich Schiller University Jena, Humboldtstraße 10, D-07743 Jena, Germany; sascha.blohm@uni-jena.de; 2 State Key Laboratory of Pulp and Paper Engineering, South China University of Technology, Guangzhou 510640, China; qihs@scut.edu.cn

**Keywords:** starch, esterification, starch formate, mixed starch ester, thermoplastic

## Abstract

Starch can be efficiently converted into the corresponding formates homogeneously using *N*-formyl imidazole obtained by the reaction of 1,1′-carbonyldiimidazole and formic acid in dimethyl sulfoxide as a solvent. Starch formates are soluble in polar aprotic solvents, not susceptible against hydrolysis, and not meltable. Thermoplastics could be generated by conversion of starch formates with long-chain fatty acids exemplified by the conversion with lauroyl chloride in *N,N*-dimethylacetamide, leading to mixed starch laurate formates. The mixed esters show melting temperatures mainly dependent on the amount of laurate ester moieties.

## 1. Introduction

Polysaccharide esters play an important role in various fields of application, e.g., as foils, films, filters, and thermoplastic to replace plastics based on fossil resources. Thermoplastic starch may be obtained by derivatisation with long-chain fatty acids that yields a disturbance of the intra- and intermolecular hydrogen bonds of the polysaccharide [1,2,3]. While polysaccharide esters of a broad variety of carboxylic acids are known and even commercially produced, the formic acid ester is scarcely investigated up until now. It was shown that organo-soluble formates of cellulose could be easily synthesised and used for esterification with other carboxylic acids [4]. Moreover, formic acid is able to dissolve carboxymethyl cellulose by acylation of the remaining hydroxyl groups [5].

Starch formate was obtained by the reaction of starch with formic acid [6,7,8,9,10,11,12,13,14,15]. However, the reaction is highly sensitive even to traces of water [8] and leads to starch formate with low average degrees of substitution (DS) only [6,7,10]. Up to now, the highest DS value of starch formate known was 2.14 and was reached at extended reaction time, high temperature, with extensively dried starch, and the use of a high excess of concentrated formic acid [8]. Unfortunately, on one hand, both formic acid chloride and -anhydride are difficult to handle under usual conditions. On the other hand, the reaction of formic acid with 1,1′-carbonyldiimidazole (CDI) has been described, resulting in *N*-formyl imidazole [16]. *N*-formyl imidazole is stable up to about 60 °C and rather sensitive to hydrolysis. Nevertheless, it is highly reactive concerning alcohols. Thus, in the present study, it was investigated to prepare a formic acid ester of starch with a high degree of substitution (DS), applying *N*-formyl imidazole. Moreover, the properties of the starch formates, including subsequent esterification, were studied.

## 2. Results

**Figure 1:**
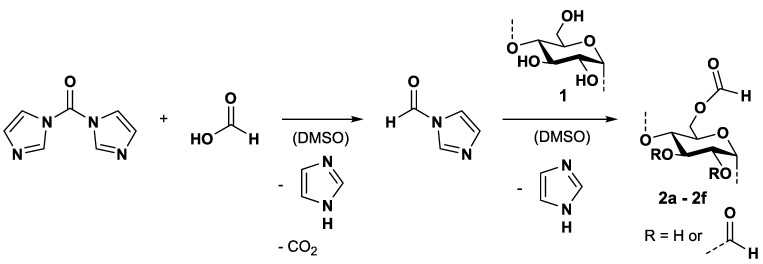
Scheme for the reaction of starch (**1**) with formic acid and 1,1′-carbonyldiimidazole yielding *N*-formyl imidazole in dimethyl sulfoxide.

A mixture of formic acid and 1,1′-carbonyldiimidazole (CDI) was allowed to react in dimethyl sulfoxide (DMSO) to form the *N*-formyl imidazole. Subsequently, the *N*-formyl imidazole was added to a solution of starch (**1**) in DMSO at an elevated temperature (Figure 1).

### 2.1. Synthesis and Characterisation of Starch Formates

Starch formate samples with degrees of substitution (DS) in the range from 0.97 to 2.55 (16 h reaction time) could be obtained by the reaction shown in Figure 1. A high DS could be realised by applying a fivefold excess of formic acid and CDI related to the anhydroglucose unit (AGU), even within a 3-h reaction time at 60 °C (Table 1). Up to now, the highest DS value of starch formate known was 2.14 and was reached at an extended reaction time, high temperature, with extensively dried starch, and the use of a high excess of concentrated formic acid [8]. Other attempts published in the literature with less concentrated formic acid or less dried starch led to a limitation of the DS of about 1.

**Figure 2:**
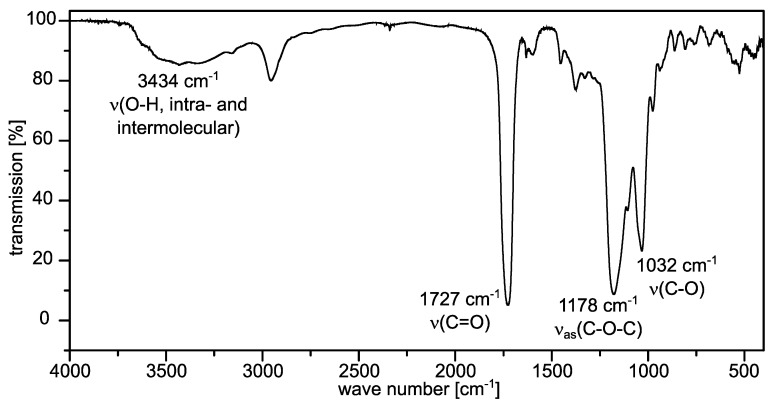
FTIR spectrum (KBr, resolution 1 cm^−1^, 64 scans) of starch formate **2d** (degree of substitution = 2.44).

The starch formates obtained in the present studies were characterised by means of FTIR- and NMR spectroscopic measurements. A typical FTIR spectrum of sample **2d**, including the signal assignments, is shown in Figure 2. The vibrational bands of the intra- and intermolecular O-H bonds are not particularly intense, which fits the high DS of the sample. On the contrary, the dominant band of the carbonyl vibration is clearly visible at 1727 cm^−1^, which is a typical value for formic acid esters [17].

**Figure 3:**
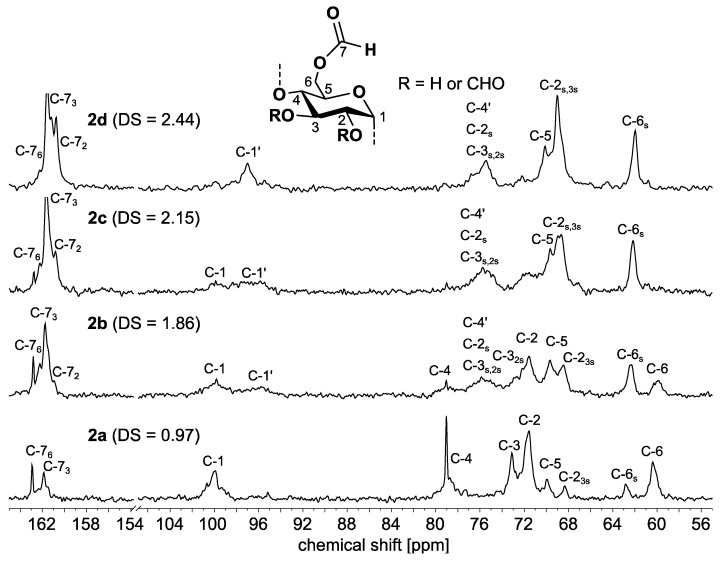
^13^C NMR spectra (62.90 MHz, 323 K, DMSO-*d*_6_) of starch formates of different degrees of substitution (DS).

The ^13^C NMR spectra of starch formate samples of different DS values are displayed in Figure 3. The signals of the AGU are found in the range of 60–100 ppm. For sample **2a** of the lowest DS value, there is only one signal visible at 99.95 ppm for position 1 of the AGU without an esterified neighboring position 2. The signal at 79.00 ppm can be assigned to position 4. The signals for carbon atoms 2 and 3 without substituents and those of carbon atom 5 can be observed at 73.11 ppm, 71.56 ppm, and 69.94 ppm, respectively. 

Position 3 with esterified position 2 adjacent is represented by the signal at 68.35 ppm. The signals at 62.85 ppm and 60.38 ppm can be assigned to position 6, which is esterified and unchanged. From these signals, it can be concluded that positions 3 and 6 of the AGU preferentially participate in the reaction with *N*-formyl imidazole. The sample **2b** with a DS of 1.86 possesses an unambiguous signal at 95.70 ppm for a carbon atom in position 1 with the neighboring esterified position 2. With increasing DS, position 6 becomes completely esterified (sample **2c**), and at a high DS of 2.44, position 2 is also almost completely esterified.

### 2.2. Properties of Starch Formates

To investigate the hydrolytic stability of the starch formates, sample **2d** was treated in a mixture of *N*,*N*-dimethylformamide (DMF) and water under stirring and the pH value was controlled continuously. The concentration was chosen to reach a pH value of 1 in the case of complete hydrolysis. Even after three hours, however, no deviation from the pH value of 7 was observed.

Independent of the DS, the starch formate samples obtained were soluble in polar aprotic solvents such as *N*,*N*-dimethylacetamide (DMAc), DMF, and DMSO. No melting could be detected for any of the starch formates at a temperature of up to 230 °C, as evaluated by a hot stage microscope. It was of interest to study the pure starch formates. It might be possible to receive a thermoplastic product if a high amount of softener is added, which would lead to a complex, as well as economical and ecological, undesired system.

### 2.3. Conversion of Starch Formate with Long-Chain Fatty Acid

**Figure 4:**
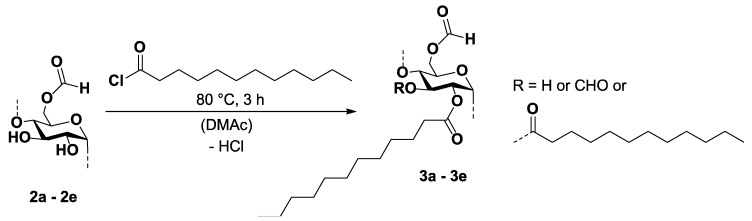
Scheme for the reaction of starch formates (**2a**–**2e**) with lauric acid chloride in *N*,*N*-dimethylacetamide.

Starch formates were allowed to react with lauric acid chloride (Figure 4). In the course of the reaction, a partial split off of formate groups occurred, which presumably could happen due to transesterification or by the chloride ions contained in the reaction medium. The nucleophilic chloride ions may attack the carbonyl group of the formic acid ester, resulting in the formation of formic acid chloride, which is not stable and readily decomposes into hydrogen chloride and carbon monoxide.

**Figure 5:**
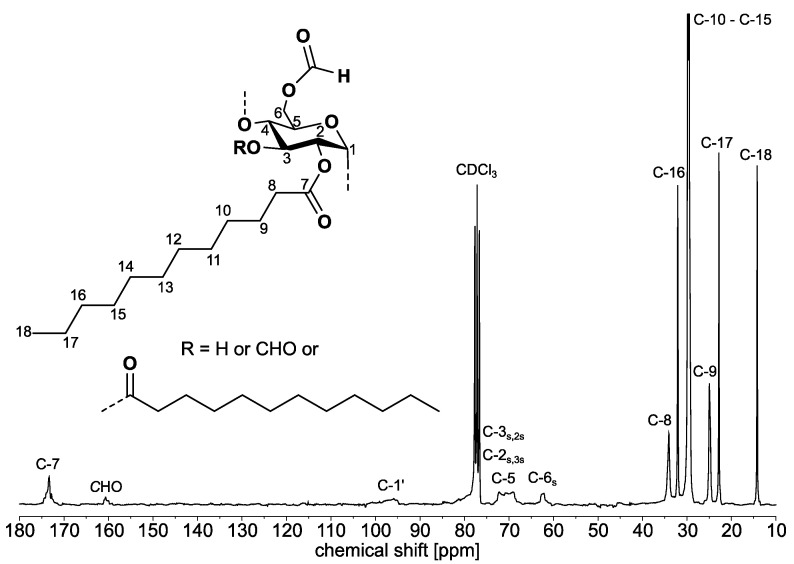
^13^C NMR spectrum (62.90 MHz, 297 K, CDCl_3_) of starch formate laurate **3a** acquired in CDCl_3_ (DS_formate_ = 0.47, DS_laurate_ = 2.53).

Samples with total DS values (DS_tot_) in the range from 1.47 to 3.00 (samples **3a**–**3d**) were accessible (Table 2). A typical ^13^C NMR spectrum of sample **3a** is shown in Figure 5. The spectrum contains the typical signals for highly substituted starch fatty acid esters. The carbon atom of the fatty acid ester causes the signal at 173.35 ppm. Furthermore, the signals of the AGU can be detected at 95.93 ppm as well as in the range from 72 ppm to 69 ppm and at 62.23 ppm. The methylene and methyl groups of the fatty acid moiety are represented by the signals at 34.06 ppm, 32.07 ppm, 29.70 ppm, 24.96 ppm, 22.81 ppm, and 14.23 ppm. Moreover, there is a signal at 160.65 ppm that can be assigned to the carbon atom of the formic acid ester.

The solubility of starch formate laurates depends on the partial DS values. A high DS_formate_ causes solubility in polar aprotic solvents such as DMF, DMAc, or DMSO. With increasing DS_laurate_, the samples become soluble in non-polar solvents such as tetrahydrofuran, acetone, ethyl acetate, and chloroform.

The starch formate laurates possess thermoplastic properties without addition of any softener. The melting ranges are slightly dependent on the total DS only; however, they strongly depend on the amount of laurate moieties (DS_laurate)_. The higher the DS_laurate,_ the lower the melting range. Sample **3d** with a DS_laurate_ of 0.11 only does not show melting at all, whereas sample **3a** with a DS_laurate_ of 2.53 melts at around 125 °C. 

## 3. Discussion

The NMR measurements revealed that the initially reactive position 3 hardly takes part in the reaction until the other two hydroxyl groups are completely esterified. Thus, positions 2 and 6 are esterified with a certain regioselectivity. The signals of the carbonyl carbon atoms are in the range of around 161 ppm. Compared to other carboxylic acid esters of starch, they are thus shifted into the high field by approximately 10 ppm.

No hydrolysis of the formic acid esters could be detected. Therefore, it can be concluded that the starch formates are more resistant to hydrolysis than previously assumed. The test could not be repeated with pure water because none of the derivatives were soluble in water due to their hydrophobic nature.

Starch formates do not possess thermoplastic properties, i.e., neither melting nor softening could be observed. However, no decomposition occurred by heating the samples up to 230 °C, which proves the thermal stability of this starch ester. On the contrary, the melting properties are governed by the fatty acid moiety, and the introduction of those long-chain moieties reduces the softening temperature remarkably. As already suspected from the investigation of the pure starch formates, the effect of the formic acid moieties is extremely weak and the formate moiety is not sufficient to generate thermoplastic starch esters.

## 4. Materials and Methods

Dimethyl sulfoxide (DMSO, 99.7%, extra dry over molecular sieve), *N*,*N*-dimethylformamide (DMF, 99.8%, extra dry), and *N,N*-dimethylacetamide (DMAc, 99.5%, extra dry over molecular sieve) were purchased from Acros Organics. 1,1′-Carbonyldiimidazole (>97%) was available from Fluka. Formic acid (>98%) was obtained from Carl Roth, Germany. Deuterated solvents were purchased from Deutero GmbH. Maize starch (FLOJEL 60, purchased from Ingredion) was dried for 8 h at 110 °C in vacuum before use.

Fourier transform infrared (FTIR) spectra were recorded as KBr pellets on a Shimadzu IRAffinity-1 (Shimadzu, Duisburg, F.R. Germany); a total of 64 measurements were accumulated with a resolution of 1 cm^−1^.

Nuclear magnetic resonance (NMR) spectra were recorded on a Bruker 250 MHz Avance I spectrometer (Billerica, MA, USA). Samples were prepared by dissolving 55 mg of the substance in 550 µL deuterated solvent. For ^13^C NMR spectra, more than 10,000 scans were accumulated. The chemical shifts are given in parts per million (ppm) and calibrated with tetramethylsilane (TMS).

The degree of substitution (DS) of formate groups was determined by applying conductometric titration after alkaline hydrolysis of the sample. For this purpose, 650 mg of the sample was added into in 25 mL sodium hydroxide solution (0.5 mol L^−1^) for 16 h at 60 °C under stirring. Aliquots of 10 mL each were taken and titrated with hydrochloric acid. The three areas of the curve were approximated with linear regressions and the intersections of the regressions gave ΔV. The DS value was calculated as the mean value of two separate measurements.

(1) $$\mathrm{wt}{\%}_{\mathrm{formate}}=\frac{\Delta V\cdot c_{\mathrm{measuring}\;\mathrm{solution}}\cdot M_{\mathrm{formate}}}{m_{\mathrm{sample}}}\cdot 100\%$$

where wt %_formate_—mass fraction of the formate substituents of the whole sample; ΔV—for titration of the formate substituent necessary volume of measuring solution in L; c_measuring solution_—substance concentration of the measurement solution in mol L^−1^; M_formate_—29.02 g mol^−1^; m_sample_—mass of the sample in g.

(2) $$DS_{\mathrm{formate}}=\frac{m_{\mathrm{AGU}}}{\frac{100\cdot M_{\mathrm{formate}}}{\mathrm{wt}{\%}_{\mathrm{formate}}}-M_{\mathrm{formate}}+1.008\;g\cdot mol^{-1}}$$

where M_AGU_—molar mass of the anhydroglucose unit (162.15 g mol^−1^).

Thermal behaviour was examined applying a hot stage microscope.

For the test of hydrolytic stability, 500 mg of **2d** (DS = 2.44, 2.17 mmol) were dissolved in a mixture of 9.54 mL *N*,*N*-dimethylformamide, and 1.06 mL deionised water. The mixture was magnetically stirred for 3 h at ambient temperature. The pH value was monitored continuously.

### 4.1. Synthesis of Starch Formate ***2a**–**2f***

In a typical example, 1,1′-carbonyldiimidazole (25.00 g, 154.19 mmol) was allowed to react with formic acid (5.82 mL, 154.19 mmol) in 91 mL DMSO for one hour at ambient temperature and, additionally, 30 min at 60 °C. The resulting clear solution was added to a solution of starch (5.00 g, 30.84 mmol) in 40 mL DMSO. The mixture was magnetically stirred for 3 h at 60 °C. After cooling to room temperature, the mixture was poured into 1000 mL chloroform. The precipitate was filtered off, washed six times with 200 mL chloroform each, and was dried in vacuum at 40 °C.

Product: **2d**

Yield: 6.58 g (28.55 mmol modified AGU, 93% of theoretical yield).

DS: 2.44 (49% efficiency).

Elemental analysis found [%]: C = 43.63, H = 4.28, N = 0.59.

^1^H NMR (250.13 MHz, 323 K, DMSO-*d*_6_) [ppm]: δ = 8.23 (-C*H*O), 5.5–3.5 (AGU).

^13^C NMR (62.90 MHz, 323 K, DMSO-*d*_6_) [ppm]: δ = 162–160 (C 7), 97.04 (C 1′), 75.53 (C 4), 72.20 (C 3_s_), 70.14 (C 2_s_), 69.02 (C 5), 61.96 (C 6_s_).

### 4.2. Synthesis of Starch Formate Laurate ***3a**–**3d***

To a solution of starch formate **2a** (1.00 g, DS: 0.97, 5.28 mmol) in 25 mL DMAc, lauroyl chloride (3.77 mL, 15.84 mmol) was added and the mixture was allowed to react for 3 h at 80 °C under stirring. After cooling to room temperature, the mixture was poured into 250 mL methanol. The precipitated formed was filtered off, washed four times with 100 mL methanol each, and was dried in vacuum at 40 °C.

Product: **3a**

Yield: 2.08 g (3.27 mmol modified AGU, 62% of theoretical yield).

DS_formate_: 0.48, DS_laurate_: 2.51.

^1^H NMR (250.13 MHz, 297 K, CDCl_3_) [ppm]: δ = 8.10 (C*H*O), 5.75–3.50 (AGU), 2.35 (H 8), 1.51 (H 9), 1.25 (H 10–H 17), 0.87 (H 18).

^13^C NMR (62.90 MHz, 297 K, CDCl_3_) [ppm]: δ = 173.37 (C 7_laurate_), 160.65 (*C*HO), 95.93 (C 1′), 72.20–68.98 (C 3_s_,2_s_, C 2_s_,3_s_, C 5), 62.31 (C 6_s_), 34.09 (C 8), 32.08 (C 16), 29.54 (C 10–C 15), 24.97 (C 9), 22.81 (C 17), 14.23 (C 18).

## 5. Conclusions

An efficient process for the preparation of pure starch formates by conversion of starch with *N*-formyl imidazole obtained by the reaction of formic acid with 1,1′-carbonyldiimidazole was developed yielding highly substituted products with DS of up to 2.55. The starch formates are soluble in polar aprotic solvents, unexpectedly stable against hdrolysis, and do not melt. However, the conversion of the starch formates by subsequent reaction with lauric acid chloride, e.g., yields mixed starch esters that are thermoplastic and melt at low temperature of 125 °C to 185 °C, depending on the DS values. The thermoplastic properties of these novel materials are under investigation.

Formate groups could act as protecting groups for the hydroxyl groups. Moreover, starch formate may act as a polymer-bound CO surrogate or CO-releasing molecule for application in organic synthesis (e. g., hydroesterification [18]) or in medical applications that are under investigation.

## Figures and Tables

**Table 1:** Conditions for and results of the reactions of starch (**1**) with *N*-formyl imidazole obtained by the conversion of formic acid and 1,1′-carbonyldiimidazole in dimethyl sulfoxide.

Sample No.	Reaction Conditions			Starch Formate	
	Temp. [°C]	Molar Ratio AGU:Formic Acid:CDI	Time [h]	DS	Efficiency [%]
**2a**	60	1:1:1	3	0.97	97
**2b**	60	1:2:2	3	1.86	93
**2c**	60	1:3:3	3	2.15	72
**2d**	60	1:5:5	3	2.44	49
**2e**	50	1:3:3	3	1.99	66
**2f**	50	1:5:5	16	2.55	51

**Table 2:** Degree of substitution (DS) and melting area of products obtained by the conversion of starch formates (**2a**–**d**) with lauroyl chloride in *N*,*N*-dimethylacetamide at 80 °C for 3 h.

Starch Formate		Molar Ratio of ModifiedAGU: Lauroyl Chloride	Starch Formate Laurate				
No.	DS		No.	DS_formate_	DS_laurate_	DS_tot_	Melting [°C]
**2a**	0.97	1:3	**3a**	0.47	2.53	3.00	125–150
**2b**	1.86	1:2	**3b**	1.31	1.15	2.46	150–175
**2c**	2.15	1:2	**3c**	1.58	0.75	2.33	160–185
**2d**	2.44	1:1	**3d**	2.51	0.11	2.62	no melting

## Data Availability

The data presented in this study are available on request from the corresponding author.

## Sample Availability

Not available.
